# An open real-time tele-stethoscopy system

**DOI:** 10.1186/1475-925X-11-57

**Published:** 2012-08-23

**Authors:** Ignacio Foche-Perez, Rodolfo Ramirez-Payba, German Hirigoyen-Emparanza, Fernando Balducci-Gonzalez, Francisco-Javier Simo-Reigadas, Joaquin Seoane-Pascual, Jaime Corral-Peñafiel, Andres Martinez-Fernandez

**Affiliations:** 1 Departamento de Teoría de la Señal y Comunicaciones, Universidad Rey Juan Carlos and Fundacion EHAS, Camino del Molino S/N, Fuenlabrada, 28943, Spain; 2 Fundación Fundatel, Mexico 366, Parana - Entre Rios, 3100, Argentina; 3 Departamento de Ingeniería Telemática”, ETSIT - Universidad Politecnica de Madrid and Fundacion EHAS, Ciudad Universitaria S/N, Madrid, 28040, Spain; 4 Servicio de Neumología and CIBER Enfermedades Respiratorias, Hospital San Pedro de Alcantara and Instituto de Salud Carlos III, Pablo Naranjo S/N, Caceres, 10003, Spain

**Keywords:** Telemedicine, Stethoscope, Tele-stethoscopy, Wireless, Real-time, E-health, Libre software, Libre hardware, Open-source

## Abstract

**Background:**

Acute respiratory infections are the leading cause of childhood mortality. The lack of physicians in rural areas of developing countries makes difficult their correct diagnosis and treatment. The staff of rural health facilities (health-care technicians) may not be qualified to distinguish respiratory diseases by auscultation. For this reason, the goal of this project is the development of a tele-stethoscopy system that allows a physician to receive real-time cardio-respiratory sounds from a remote auscultation, as well as video images showing where the technician is placing the stethoscope on the patient’s body.

**Methods:**

A real-time wireless stethoscopy system was designed. The initial requirements were: 1) The system must send audio and video synchronously over IP networks, not requiring an Internet connection; 2) It must preserve the quality of cardiorespiratory sounds, allowing to adapt the binaural pieces and the chestpiece of standard stethoscopes, and; 3) Cardiorespiratory sounds should be recordable at both sides of the communication. In order to verify the diagnostic capacity of the system, a clinical validation with eight specialists has been designed. In a preliminary test, twelve patients have been auscultated by all the physicians using the tele-stethoscopy system, versus a local auscultation using traditional stethoscope. The system must allow listen the cardiac (systolic and diastolic murmurs, gallop sound, arrhythmias) and respiratory (rhonchi, rales and crepitations, wheeze, diminished and bronchial breath sounds, pleural friction rub) sounds.

**Results:**

The design, development and initial validation of the real-time wireless tele-stethoscopy system are described in detail. The system was conceived from scratch as open-source, low-cost and designed in such a way that many universities and small local companies in developing countries may manufacture it. Only free open-source software has been used in order to minimize manufacturing costs and look for alliances to support its improvement and adaptation. The microcontroller firmware code, the computer software code and the PCB schematics are available for free download in a subversion repository hosted in SourceForge.

**Conclusions:**

It has been shown that real-time tele-stethoscopy, together with a videoconference system that allows a remote specialist to oversee the auscultation, may be a very helpful tool in rural areas of developing countries.

## Abstract (es)

**Abstract:**

**IntroducciÃ³n**

Las infecciones respiratorias agudas son la principal causa de mortalidad infantil. La falta de médicos en las zonas rurales de países en desarrollo hace difícil su correcto diagnóstico y tratamiento. El personal que atiende los puestos de salud rurales (técnicos de salud) no están formados para distinguir las enfermedades respiratorias por auscultación. Por esta razón, este proyecto propone el desarrollo de un sistema de tele-estetoscopia que permita a los médicos recibir en tiempo real los sonidos cardiorespiratorios de una auscultación que se está produciendo en un lugar remoto , así como ver la imagen del técnico para saber dónde está colocando el estetoscopio en el cuerpo del paciente.

**MÃ©todos**

Se ha diseñado un estetoscopio inalámbrico de tiempo real. Los requerimientos iniciales fueron: 1) El sistema debe de enviar el audio y el vídeo de forma sincronizada, sobre redes IP, no requiriendo una conexión a Internet; 2) Se debe de preservar la calidad de los sonidos cardiorespiratorios, permitiendo adaptar la campana y las olivas de un estetoscopio convencional, y; 3) Los sonidos cardiorespiratorios deberían de poder ser grabados a ambos lados de la comunicación. Para verificar la capacidad diagnóstica del sistema se diseñó una validación clínica con ocho especialistas. En un estudio preliminar, cada uno de los ocho médicos auscultó a doce pacientes, utilizando tanto el sistema de tele-estetoscopia como un estetoscopio convencional. El sistema debe de permitir escuchar los sonidos cardiacos (murmullos diastólicos y sistólicos, galope, arritmias) y los respiratorios (roncus, estertores crepitantes, sibilancias, sonidos bronquiales atenuados y roce pleural).

**Resultados**

Se describe en profundidad el diseño, la implementación y la evaluación inicial de un sistema inalámbrico de tiempo real de tele-estetoscopia. El sistema fue concebido con filosofía de código abierto y bajo coste, con a intención de que otras universidades y/o pequeñas empresas pudieran fabricarlo. Se ha utilizado únicamente software abierto para reducir los costes de fabricación y para buscar alianzas futuras para su mejora y adaptación. El firmware de microprocesador, el software del ordenador y los planos de la PCB están accesibles para su descarga en un repositorio de SourceForge.

**Conclusiones**

Se ha podido mostrar que un sistema de tele-estetoscopia en tiempo real, unido a un sistema de videoconferencia que permita a los especialistas realizar la auscultación a distancia, puede ser una herramienta muy útil en zonas rurales de países en desarrollo.

## Background

### Introduction

In 2000, the United Nations settled the Millennium Development Goals (MDG), engaging all governments in a common effort to improve development indicators. According to the MDG, the mortality rate of children under 5 years should drop to 30 deaths per 1,000 live births by 2015. This rate doubled the target in 2009, and the specific rate for Subsaharian Africa was five times greater than the target in 2008. The maternal mortality rate, which was not supposed to exceed 120 deaths per 100,000 live births, will exceed 600 in Africa [1,2]. Unfortunately, we can say that healthcare in developing countries is a world-class problem, for which solutions must be found by all means available, including the use of telecommunications and telemedicine.

The Spanish EHAS Foundation and the Argentinian Fundatel focused on this reality years ago and started to work on improving healthcare processes through the use of ICT in rural areas of developing countries. The proliferation of appropriate wireless IP telecommunications networks in rural areas [3] is promoting the research on telemedicine services according to that reality.

The main cause of morbidity and mortality in children under 5 in rural areas of developing countries are ARI (acute respiratory infections). The lack of physicians makes it difficult to reach the correct diagnosis and treatment of these diseases. Health care staff at these facilities may not be qualified to distinguish respiratory diseases by auscultation [4]. For this reason, this project proposes the development of a tele-stethoscopy system that will allow the physician to receive real-time cardio-respiratory sounds from a remote auscultation, as well as video images showing where the technician is placing the stethoscope on the patient’s body. The aim was to develop a tool that would bring the doctor virtually to the isolated rural area, using free software and hardware in order to reduce future manufacturing costs. The project was funded by the Universidad Politecnica de Madrid and Universidad Rey Juan Carlos, and executed by EHAS and Fundatel Foundations with support from the Pulmonology Department at Hospital San Pedro de Alcantara (Caceres) which provided clinical validation of the system.

### Cardiorespiratory signal and A/D conversion

The stethoscope is a device that allows an operator to listen cardiorespiratory and intestinal sounds, as well as blood flow in vessels.

It is constituted by:

• Chestpiece, a double-sided element that captures sounds from the patient’s body. The bell is located at one side and the diaphragm at the opposite.

• Acoustic tubes, allow sound transfer from the chestpiece to binaural pieces.

• Binaural pieces or ear tubes, two metallic elements that bring the sound from the acoustic tubes to each of the earpieces.

• Earpieces, devices that adapt the binaural pieces to the ears.

The sounds captured by a stethoscope have particular spectral characteristics and structure [5]:

Heart sounds (20-600 Hz):

• The first heart sound is in the 20-115 Hz range (including pathological sounds).

• The rest of heart sounds are at the 140 Hz - 600 Hz range.

Respiratory sounds (50-1200 Hz):

• The most important part of the signal is below 100 Hz, although useful components may be found up to 1.2 kHz.

This frequency distribution requires careful consideration regarding audio capture and A/D (analog-to-digital) conversion. The fact that most of components of the respiratory signal are below 100 Hz, any hardware unable to scan these frequencies may degrade quality enough to make the system unsuitable for medical diagnosis.

### State of the art

Electronic stethoscopy is not a new concept. There are many commercially available digital stethoscopes that send, store and analyze sound via computers, helping in the diagnosis [6]. Although some electronic stethoscopes perform well enough t [7,8] to record and reproduce high-quality audio, most work locally, connected to a computer via data cable. An interesting contribution is [9], adding videoconference to audio transmission, but requiring a data cable connected to a separate WiFi device. Finally we must cite [10] that sends the sound from the stethoscope to a PDA using bluetooth in order to show the phonocardiogram to the patient.

The patent databases have been reviewed in order to determine the existence of previous systems similar to ours. All combinations of the keywords electronic/wireless/digital stethoscope were used in our queries. After reviewing more than 170 patents, no coincident results were found. On the other hand, were found electronic stethoscopes that convert acoustic to electric signals for better local processing, for example patents WO0232313 and WO2009155593 (filed in 2002 and 2009 respectively). Some wireless stethoscopes which convert the signal to digital form and send it locally to the headphones were found (WO2008097008, 2008), allowing both , doctor and patient, to listen cardiorespiratory sounds at the same time. None of these systems allow real-time tele-stethoscopy. Several over the phone stethoscopes were found, for example US20060018278 in 2006, or WO2008097009 in 2008, which allow remote diagnosis of cardiorespiratory diseases. These systems are limited to the transmission of data and sound through phone lines, and do not allow send both audio and video over IP networks as our does, which is essential for real-time guidance to the technician performing the exam. The generic US Patent 7115102 (2006) describes a real-time stethoscopy system but without transmission either over IP networks or wireless capabilities.

“3M Littman Scope to Scope Teleauscultation System” recently became commercially available, sharing many functional capabilities with our system. It allows only unidirectional real-time wireless transmission with no videoconferencing system integrated. In addition of real time visualization of the remote auscultation, our system presents two fundamental differences when compared to the mentioned system:

• Our system is a real-time wireless stethoscopy system which can be adapted to any commercially traditional available stethoscope (transforming it into a tele-stethoscope) . This feature allows health care providers to use the mechanical parts of their own stethoscopes.

• This system is conceived from scratch as open-source and low-cost device. It is designed in such a way that local universities and small companies in developing countries may manufacture it, strongly conditioning design and choice of components.

## Methods

**Figure 1:**
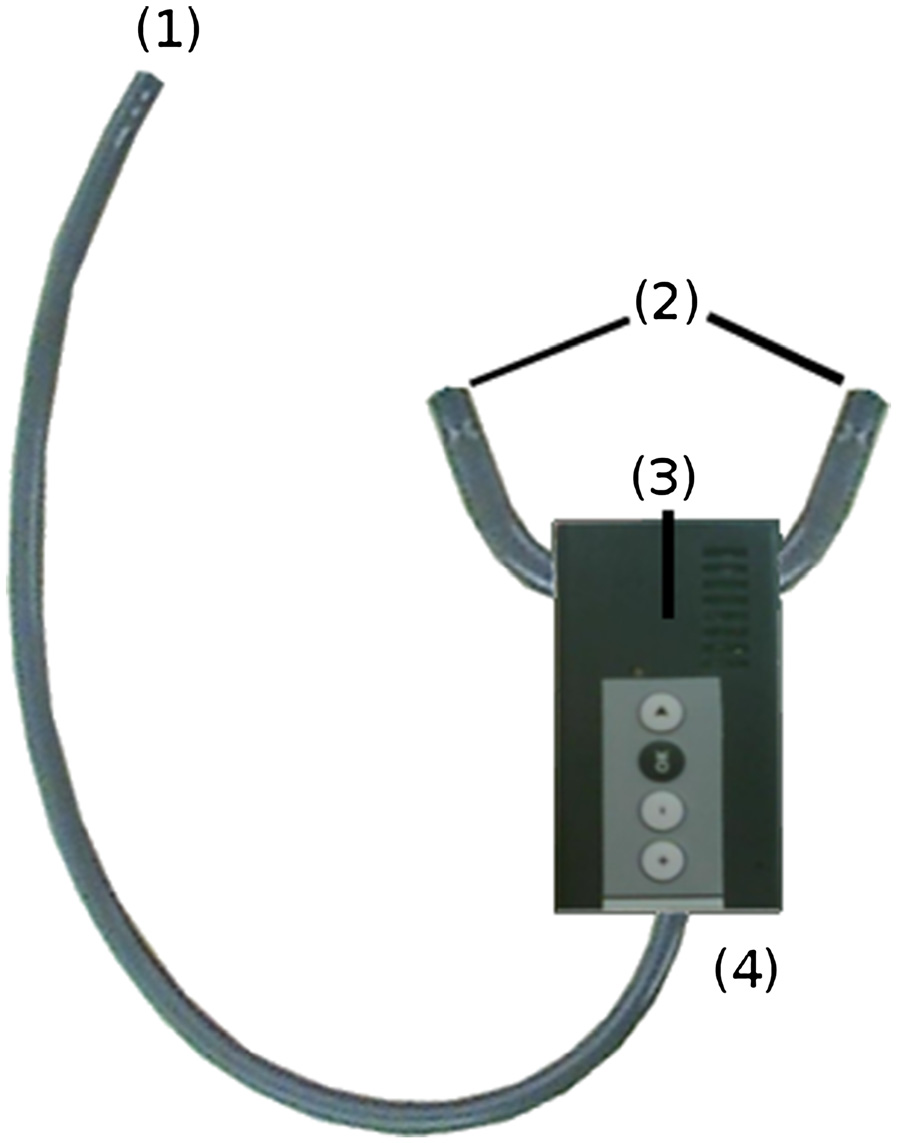
“EHAS-Fundatel digital stethoscope”.

The main objective of the project is the design and development of a real-time wireless stethoscopy system that sends audio and video synchronously over IP networks. The system must preserve the quality of cardiorespiratory sounds, and allowing to adapt the binaural pieces and the chestpiece preferred by the user at each end, so that the whole stethoscopy system must keep the mechanical characteristics of the stethoscopes previously used by the health care providers (see Figure 1). Other important requirements that influence the design are:

• The remote doctor must obtain a similar experience when using our system or when using a conventional stethoscope.

• Audio and video from auscultation must be synchronized and function properly over a data network, not requiring an Internet connection.

• Cardiorespiratory sounds should be able to be recorded at both sides of the communication in order to allow retrospect interpretation and clinical audits.

• Quality must be at least as good as for a conventional stethoscope, which requires technical and other clinical validation.

The technical validation includes tests to verify the spectral response in the range of clinical interest and the real time bidirectional audio integrity. 8 clinicians (4 pneumologists, 2 cardiologists and 2 internists) were responsible for a preliminary diagnostic capability validation of the system. Patients were examined both remotely and locally by all 8 physicians. Local exams with an standard stethoscope were considered the gold standard. The sample size (twelve for the preliminary analysis and forty for the whole trial) has been selected to evaluate its capacity to listen the main cardiac (systolic and diastolic murmurs and location, murmur over the carotids, gallop sound, arrhythmias) and respiratory (rhonchi, rales or crepitations, wheeze, diminished and bronchial breath sounds, pleural friction rub) sounds.

Written informed consent was obtained from all participants of the study for publication of this paper including accompanying images. A copy of the written consent is available for review by the Editor-in-Chief of this journal.

## Results and discussion

### Description of the system developed

The system consists of a stethoscopy tubing which allows connection to custom binaural pieces and a chestpiece, with electronics contained in a central cabinet (see Figure 1).

**Figure 2:**
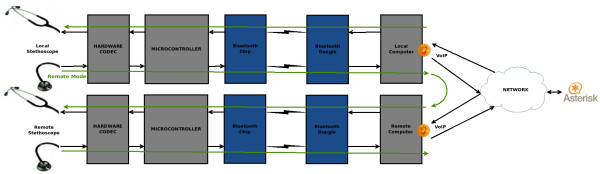
“Block diagram of remote operation mode”.

When the sound is captured by the chestpiece connected to (1), a microphone in (4) transfers analog audio to a hardware codec that performs the A/D conversion. Once the signal has been digitally converted, a microcontroller sends the audio in two alternate operation modes:

• In local mode the microcontroller sends the audio back to the codec and performs a D/A conversion, and send it finally to embedded headphones in (3). The sound is transferred to the earpieces through binaural pieces connected to (2). This mode emulates the way conventional stethoscopes work.

• In remote mode (see Figure 2), the microcontroller sends the audio signal both to the local loop and to a bluetooth chip that forwards the sound to a local computer. The wireless communication incorporates a flow control mechanism that assures audio integrity.

When the digital sound reaches the computer, a software package decodes the audio signal and this is forwarded to two alternate destinations:

• Local computer’s soundcard. This allows real-time sound playback, useful for diagnosis if the soundcard has an adequate frequency response. It also allows local storage into the patient’s digital records, additional processing and forwarding. These features allow a second opinion as well a legal support if necessary.

• VoIP client application. Our system was currently validated for Ekiga 3.2 and higher versions, allowing real-time audio and video transmission from a webcam over an IP communications network. The final destinations will be specialists in a remote location, or medical students for teaching.

This software package is currently only working for GNU/Linux, but we are in the process of develop versions for other operating systems.

The VoIP software codes both audio and video signals, using a software codec that optimizes the bandwidth use. On the other end, the receiving computer applies the inverse procedure, recovering audio and video signals with a quality that depends on the codec used.

The VoIP software allows audio and video signals be sent to any computer or group of computers connected via Internet. This feature allows the auscultation process be monitored and controlled remotely by the doctor.

Audio signals can be redirectioned in the remote computer to the following destinations:

• A second digital tele-stethoscope similar to the one used for examining the patient, allowing the user real time analysis of the captured sound at the other end. This destination is the recommended in telemedicine.

• The soundcard allowing store, processing and listen through external speakers or headphones connected (this destination is conditioned to adequate frequency response at all these components).

The remote digital tele-stethoscope receives the audio from the remote computer via the bluetooth link, rebuilds the audio signal and sends it to the hardware codec for D/A conversion. The sound is then reproduced and sent to the binaural pieces with identical performance to that of the sending stethoscope. The whole process occurs in real-time and bidirectionally.

The goal of the tele-stethoscopy system presented is to solve the problem of rural health facilities in developing countries where doctors are not available. The system will allow doctor to guide a local technician remotely via videoconference, as well as to listen to cardiorespiratory sounds for clinical diagnosis. This will improve diagnosis, reduce the start of treatment, eliminate the need for the patient to travel long distances and, consequently, reduce costs for both patient and the healthcare system.

#### Hardware development

The mechanical characteristics of standard stethoscopes have been taken into account for the design of the proposed system in order to assure optimal acoustic quality. Some of the concerns in this step of the design have been:

1) The chestpiece is a filter itself that separates adequately the cardiorespiratory sound from environmental noise. Hence, the design of the chestpiece is not altered, only a microphone is introduce with an appropriate frequency response to capture the sound and transform it into an electrical signal.

2) The microphone is situated strategically in the acoustic pathway inside the flexible tubing, allowing the user to adapt the preferred chestpiece. The same concern determined the position for the embedded speaker, which is located inside the piece of tubing prior to the bifurcation so that any binaural pieces may be adapted.

These design requirements assure the ergonomy of the final device, and guarantee that the user experience is kept very similar to that of conventional stethoscopes. All the electronic circuits and devices are inside a central cabinet in which the system concentrates the interaction user ↔ device.

##### Steps

**Figure 3:**
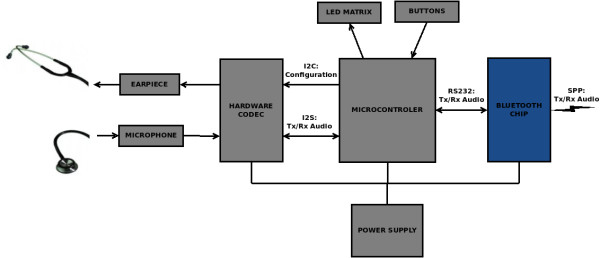
“Block diagram of the EHAS-FUNDATEL ditial tele-stethoscope hardware”.

The elements of EHAS-Fundatel tele-stethoscopy system are (see Figure 3):

• Microphone.

• Embedded speaker.

• Hardware codec.

• Microcontroller.

• Bluetooth chip.

• LED matrix.

• Control buttons.

• Power supply.

A description of the main elements’ characteristics follows.

##### Microphone and embedded speaker

These are essential elements in the acoustic performance of the final device. Their quality affects directly to the audio quality perceived by the user. Their main requirement is their bandwidth, which must be respectful between 20Hz and 1200 Hz.

The microphone used is the omnidirectional model Panasonic WM-64PR, which has a sensitivity of -45 dB, a frequency operating range between 20 Hz and 16000 Hz and a SNR over 58 dB. This microphone is especially suitable because of its small size and great resistance to RF noise.

The speaker was taken from Philips headphones model SHE9500. It has an impedance of 16Ω, a sensitivity of 102 dB and a frequency range between 6 Hz and 23500 Hz.

##### Hardware codec - TLV320AIC33

This audio codec possesses the advantage of having differential inputs, high fidelity, low power consumption and pseudo-stereo output -this last feature is not used in our current version-. Other reasons considered for this choice were:

• The DAC converter SNR is 100 dB, and the ADC converter SNR is 90 dB.

• It has an I2S interface for digital audio, which facilitates and improves the audio transmission to the microcontroller.

• It has 10 inputs and 7 outputs, which gives many options for future extensions.

• The operation temperature covers a range from -45 up to 85°C, sufficient for operating within any region in the world.

• Initially the sampling was fixed to 16 bits per sample, but the codec may use up to 24 bits per sample if necessary.

##### Microcontroller - dsPIC30F4013

A hybrid microcontroller + DSP has been chosen from the dsPIC30F family, containing a data conversion interface (DCI) including the I2S audio protocol. The fact that the hardware codec also supports this protocol makes audio exchange easier.

The following arguments have supported the choice of this dsPIC:

• High processing power, the kernel is designed to run high-level digital audio filtering algorithms.

• Low power consumption and high speed.

• Many general purpose peripherals and some specific for audio, as well as several memory options.

The role of the dsPIC in this design is to manage the digital audio received and to forward it in real time to the hardware codec and/or the bluetooth interface, always preserving the sound quality and filtering noise.

##### Bluetooth chip - OEMSPA310

The bluetooth interface exchanges digital audio data with the computer at a bitrate of 460800 bps. Provided that the digital sampling at the audio codec generates 128 kbps (sampling at 8 kHz with 16 bits/sample), and the control protocol used over the bluetooth link converts each 16 bits sample to 24 bits, the total one-sense data rate is 192 kbps. Therefore, the chip is fast enough to send and receive real time audio simultaneously.

##### Power supply module - TPS61107PWR

The element used provides with a complete solution for energy management in battery-powered devices. It works with input voltages as low as 0.8 V in order to maximize battery life.

##### PCB

A doubled-sided PCB has been designed because this technology may be used in most developing countries. The shape is conceived to allow the central cabinet be comfortably placed under the binaural pieces.

#### Software development

There are two software subprojects in the design: firmware for the electronics and software for the computer.

##### Firmware

The operation of the device is controlled by a main program run by the microcontroller, which in turn commands the other modules:

• LEDS → Controls the LED matrix.

• RS232 → Initialization and control of the UART2 at the dsPIC.

• BTATCommands → API for controlling bluetooth AT commands.

• Coding → Introduces control characters into audio samples received from the hardware codec.

• DCI → Configures and manages the I2S bus, which is used for digital audio communication between microcontroller and codec.

• I2C → Provides the codec with the initial configuration that allows to work with the I2C bus.

• Buttons → Configures interruptions for buttons.

• Timer → Configures the timer at the microcontroller.

• Several Functions → Controls standby operation functions required for configuration processes.

##### Computer software

The computer plays an important role in the tele-stethoscopy system. In future versions the computer could potentially be replaced by a smartphone. Several programs synchronize the audio transmission or reception together with a standard videoconference system.

### Audio subsystem

The most extended sound driver for GNU/Linux is ALSA, which enables the operating system to control the hardware. A sound mixer is also needed, and Pulseaudio has been our choice. This is a multi-platform sound server with the following features:

• Independent volume control for each application.

• Extensible architecture supporting dynamic modules.

• Compatible with most audio applications.

• Supports multiple audio sources.

• Low-lattency operation and lattency monitoring.

• Zero-Copy memory architecture, for improved processor performance.

• Self-discovery of peer Pulseaudio systems in the local area network, and local reproduction to the speakers.

• Possibility of hot-swapping the audio output device while the audio is being reproduced.

• Command-line interface with scripting functionalities.

• Recoding and sampling functionalities.

• Dynamic detection of audio bluetooth devices.

This is a truly real time system. Real time sessions can also be stored while the system is running by using Audacity application. Recording the session with Audacity doesn’t interfere with the real-time process and also allows the user to play back recorded sounds at a later time. It also allows received audio processing and session archive for future support in case of legal conflict. A Jack connection kit has been used to allow the system to send audio signals to different destinations (such as to Audacity and a remote stethoscope at the same time). Jack is a very powerful audio switch among applications, allowing several real-time operations. Jack can work directly with the ALSA driver and Pulseaudio. Jack_Rack has also been used to apply bandpass filtering in real-time. This gives the possibility to isolate certain spectral components of the cardiorespiratory signal.

Hence, the audio subsystem is finally made up with the following elements:

• ALSA backend driver.

• Pulseaudio sound server.

• Jack connection kit for audio switching among sound clients.

• Audacity for recording and analysing audio.

• Jack_Rack for real-time audio filtering.

### Softphone

Conventional VoIP software has been used for audio and video exchange among computers. In the selection of VoIP tools, future sustainability has been a must.

The most extended, supported and well-tested open-source software PBX that meets the requirements is Asterisk.

The selected client application has been Ekiga. However, other alternatives can be easily considered.

### Hardware interconnection software

**Figure 4:**
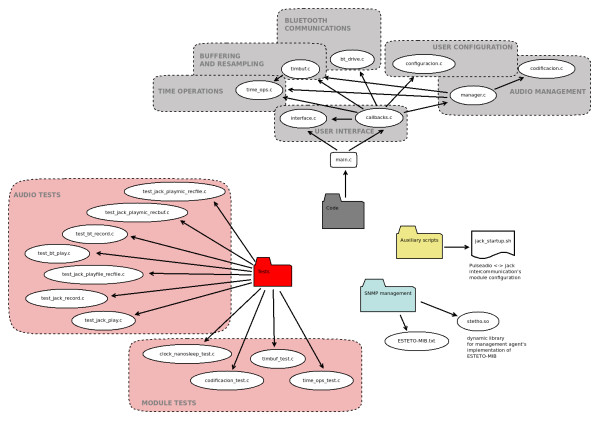
“PC software modular structure”.

The resident application that controls the stethoscopy audio subsystem in the computer is modular. Figure 4 shows the structure of the code.

**Figure 5:**
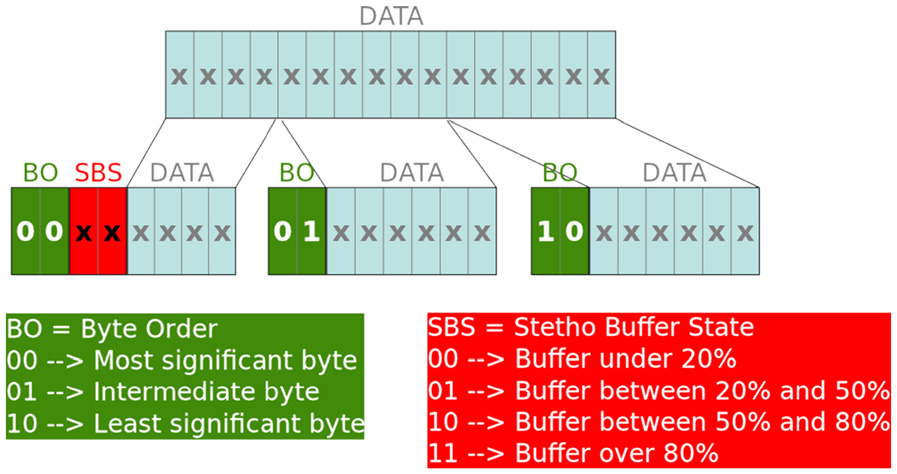
“Bluetooth audio protocol”.

**Figure 6:**
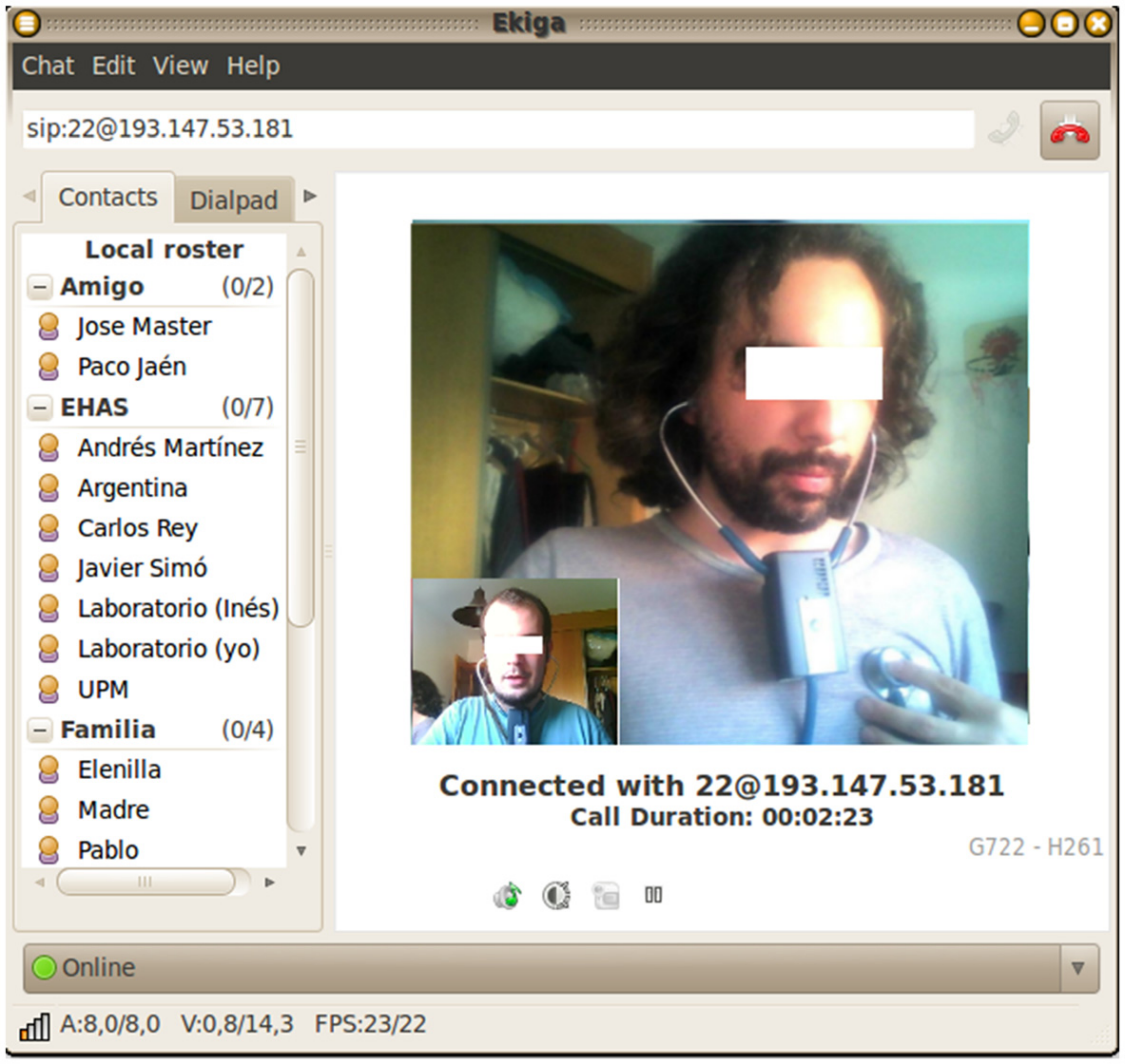
“Graphic interface of the tele-stethoscope software”.

The modules are:

• Bluetooth communication. It carries out basic operations including bluetooth connections setup and search for devices.

• Real-time audio storage and resampling. We have designed and developed a bidirectional storage and resampling module that dynamically keeps the tele-stethoscopy system (working at 8 KHz) synchronized with any soundcard at any sampling frequency used. The module self-calibrates during the first 8 seconds. It then receives audio signal from the soundcard, detects its operational frequency and starts adapting the signal to the hardware codec frequency. In order to avoid sample losses due to the lack of synchronization between the computer and the sthetoscope, a control system called SBS (Stetho Buffer State) has been introduced. This system slightly increases or decreases input and output data flows depending on how the system is working. Figure 5 shows how the buffer status is reported in every data packet with two bits that code whether the buffer is under 20% of its capacity, between 20% and 50%, between 50% and 80%, or over 80%.

• Audio management. It performs setup and surveillance of sound server’s callbacks, exchanges audio data with the bluetooth interface using the resampling library for storing and extracting the audio.

• Graphic Interface. This module has an intuitive graphic interface with a few buttons and texts (see Figure 6). It interacts with the other software packages through modules that manage the callbacks generated by each event at the interface.

• Temporary operations. Performs time management functions for the rest of the software.

• Configuration. Guarantees that certain user-defined parameters may be reloaded in future sessions.

**Figure 7:**
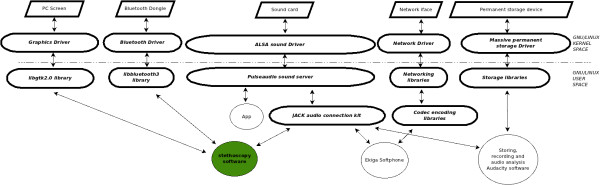
“Software architecture of the digital tele-stethoscope”.

The software architecture is detailed in Figure 7.

### Technical and clinical validation

Several test series were conducted in order to verify that bidirectional audio is sent and received accurately in real-time. The first test attempted to confirm that the microcontroller could manage processing and forward signals simultaneously. The second test checked the memory management. The third test series aimed to verify signal integrity at each system stage: coding and decoding, wireless links between stethoscopes and computers, audio subsystems and IP internetwork. A detailed description for each test series follows:

• Microcontroller Tests: Echo tests with sinusoids generated at the computer aimed to compare sample by sample signals sent from the computer and received back. The stethoscopes were programmed for echoing each sample for this tests and the resampling module was turned off in order to isolate possible interferences with the microcontroller from others due to buffering and resampling. The system response was completely accurate in terms of amplitude, frequency and phase for all the cases, no sample loss was detected, thereby showing that the controller is fast enough to manage bidirectional real-time audio.

• Memory Management Tests: The way a storage & resampling module installed in the PC synchronizes the stethoscope with the computer’s soundcard has been previously described. This subsystem must monitor microcontroller’s memory to prevent it from becoming empty or saturated due to lack of synchronization. The microcontroller was programmed to send an error signal when the buffer was under 20% or over 80%. During the tests, the error signal was never issued.

• Coding and Decoding Tests: Processes were tested by sending sinusoids with amplitudes fitting in all quantization steps. Other similar tests were run to verify signal integrity through the whole system, with checkpoints at each software module as well as at each end of wireless links.

Prior to clinical validation, a technical validation test was performed in order to verify system frequency response. Pure tones at 10-1500 Hz frequency range were generated, captured by the first stethoscope microphone and sent through the entire system chain to the second stethoscope. A digital oscilloscope captured the audio from the output of the second stethoscope and flat response at 20-1200Hz frequency range was verified. The system had good specifications even at lower frequencies.

Finally, there have been several subjective tests with cardiologists and pulmonologists in Spain and Peru. In the first trials, we experienced some problems with regards to excessive end-to-end latency and excessive noise produced by the contact of the chestpiece with the patient’s clothing. Those problems were solved improving the code that manages the resampling module, adjusting the microphone gain and upgrading the G.711 codec to the G.722 codec.

A preliminary study with only twelve patients (a larger clinical trial is ongoing and the testing and bechmarking results will be tentatively publisher in 2013) was performed in Spain. Eight doctors (four pulmonologists, two cardiologists and two internists), examined the twelve patients. Doctors were blind to patients’ medical records and could not discuss exam findings. Patients were randomized in two different groups: standard auscultation (at patient’s bedside) and telematic auscultation (through the hospital intranet). All the cardiac and respiratory auscultations during sessions were made similarly, according to a map of auscultation which included six cardiac and twelve respiratory sites of auscultation. All the cardiac and respiratory auscultations during sessions were made similarly, according to a map of auscultation which included six cardiac and twelve respiratory sites of auscultation. Cardiac examination included: heart rate, systolic or diastolic murmur, gallop sound and other murmurs. Respiratory examination included: decreased or preserved breath sounds, rhonchi, wheezing and crackles.

The aims of this preliminary study were as follows: 1) to evaluate the acoustic quality of our real-time wireless tele-stethoscopy system; 2) to test this system with different medical specialists on different patients and spectrum of disorders (lung, heart and infectious diseases); and 3) to evaluate qualitative differences and concordances (the quantitative study will be finished in 2013) among different medical doctors. The main results were:

• The acoustic quality of the system was compared with a standard stethoscope (Classic II-Littmann). The main reported differences were fine noise at the bottom and excessive delay in signal initially (both reported problems were finally solved by the engineering team). Using a five level acoustic quality scale (very good, good, regular, bad and very bad), 87,5% of the doctors reported either good or very good acoustic quality.

• The inter-observer agreement was good (≥ 6 medical doctors) for diminished breath sounds, rhonchi and heart rate with 2 patients of COPD; very good (8) for heart rate, crackles and systolic murmurs for 2 patients with heart failure; good for rhonchi or wheezing with 1 patient with asthma; good for heart rate with the patient with auricular fibrillation but poor (4) for systolic or diastolic murmur; good for heart rate, systolic murmur and crackles for 1 patient with myocardial infarction; good agreement in heart rate, systolic murmur, rhonchi or wheezing and crackles, but poor agreement in diminished breath sounds with 1 patient with respiratory failure; good in heart rate, systolic murmur and lung auscultation but poor in diastolic murmur for 1 patient with valvular disease; very good agreement in all the auscultation of 1 patient with lung cancer; good in the lung examination for 1 patient with myasthenia gravis; and good agreement in diminished breath sounds, crackles, rhonchi, heart rate and systolic murmur for 1 patient with pneumonia. As expected, we found better agreement among physicians with same medical speciality and similar degree of experience.

• Our preliminary results show intraobsever agreement (degree of agreement of one doctor using both methods) in eight out of twelve patients for all respiratory and cardiac sounds.

• The average exam time for tele-auscultation was 191 seconds and the average exam time for standard auscultation was 110 seconds.

By the middle of 2013, a clinical trial with 40 patients with cardiac and respiratory diseases (sample size was calculated according to the higher prevalence of adventitious lung and cardiac sounds) will be completed in Spain with the aim to obtain sensitivity and specificity of the system. The results of this trial will be tentatively published at a later time.

In Peru, the system was tested by two cardiologists and pulmonologists in a real telemedicine scenario between Santa Clotilde health center and Loreto Regional Hospital. This was conducted over a 180 Km long wireless network. The results of these tests were satisfactory in all cases.

### Software and hardware schematics download

Only free open-source software has been used in order to minimize manufacturing costs and search for alliances which may lead to future improvements. The description of the licenses follows:

• Pulseaudio sound server → GPLv2.

• Jack connections kit → The program uses GPLv2, but the library uses LGPL.

• Ekiga audio client → The program uses GPLv2, but links to libraries such as OPAL, OPENH323 or PWLIB with different licenses that have been included in the GPLv2 after asking for permission to the developers. The libraries’ licenses are MPLv1, which is also free software but requires authorization for compatibility with GPLv2.

• Tele-stethoscopy program → GPLv3.

• G.722 codec → The patent expired several months ago, now it is in the public domain.

• Asterisk PBX → It uses a GPLv2 license.

The composition of all these licenses results in a whole free open-source software system.

The microcontroller firmware code, the computer software code and the PCB schematics (including the list of required components) are available for free download in a subversion repository hosted in SourceForge [11].

## Conclusions

This paper justifies the need for supporting healthcare at isolated rural health facilities in developing countries in regards to respiratory infections diagnosis. It has been shown that real time tele-stethoscopy, along with a videoconference system which allows a remote specialist to oversee the auscultation, may be a very helpful tool. The paper has described in detail the design, development and initial validation of a real-time wireless tele-stethoscopy system created from scratch with open-source software, hardware and knowledge. The formal clinical validation trials will be held by the end of 2012. The results, positive or negative, will be sent for publication as soon as possible (2013).

Beyond the motivating application for tele-stethoscopy, medical training is another important scenario in which this system can be useful. This system allows a teaching physician to hold the stethoscope on a patient and share that exact sound with an unlimited number of students. The potential of this product is not limited to students on site given, the sounds the doctor hears can be transmitted anywhere via Internet. This portability offers exceptional educational capabilities.

The main contribution of this paper is sharing the potential of this concept with the scientific community and the manufacturers of tools, programs, schematics, scripts for technical validation, etc. This information will enable anyone to build prototypes that can be further improved, and even marketed. The cost of materials for a prototype is as low as 170 USD, hence industrial production may be done at a very low cost. New technical and clinical validation trials from the scientific community are welcome, especially trials in real scenarios.

This system was initially designed to be used in small clinics in rural areas where in-built computer bluethooth coverage should be enough to cover the entire area. If the system was to be used in larger spaces such as hospitals, a bedside bulky computer should not be required. In such a clinical scenario, the stethoscope can be connected via bluetooth to a WiFi -3G smartphone with video-conference capabilities. The system migration to Android based cellphones, a work in progress by our developer team, should be relatively simple, as these use a Linux based operating system.

## Abbreviations

IP: Internet protocol; PCB: Printed circuit board; MDG: Millennium development goals; EHAS: Enlace Hispano Americano de Salud (Hispanic American Health Link); ICT: Information and communication technologies; ARI: Acute respiratory infections; Hz: Hertz; A/D: Analog-to-digital; D/A: Digital-to-analog; WiFi: Wireless fidelity; PDA: Personal digital assistant; VoIP: Voice over internet protocol; GNU: GNU is not unix; LED: Light-emitting diode; SNR: Signal to noise ratio; dB: Decibel; RF: Radio frequency; DAC: Digital analog converter; I2S: Inter-IC sound; DSP: Digital signal processor; API: Application programming interface; DCI: Data conversion interface; ALSA: Advanced linux sound architecture; PBX: Private branch exchange (private telephone switchboard); SBS: Stetho buffer state; GPL: General public license.

## Competing interests

The authors declare that they have no competing interests.

## Authors’ contributions

RRP and GHE developed the hardware and the microcontroller firmware code . IFP, JSP and FJSR developed the computer software code. AMF and FBG conceived the project, and participated in its design and coordination and helped to draft the manuscript. JCP carried out the clinical trials. All authors read and approved the final manuscript.
